# Enhancement of Weld Penetration via Arc Constriction in 316L Stainless Steel Using High-Frequency Flat-Top Longitudinal Magnetic Field-Assisted TIG Welding

**DOI:** 10.3390/ma19102128

**Published:** 2026-05-19

**Authors:** Yingzhe Liu, Hongfa Ding, Jian Luo, Chenhe Chang, Lina Zhao, Yunlong Chang

**Affiliations:** 1 Wuhan National High Magnetic Field Center, Huazhong University of Science and Technology, Wuhan 430074, China; liuyingzhe@hust.edu.cn; 2 School of Materials Science and Engineering, Shanghai University of Engineering Science, Shanghai 201620, China; luojian@sues.edu.cn; 3 Liaoning Xinyuan Special Welding Technology Co., Ltd., Shenyang 110011, China; xychangch@126.com; 4 School of Materials Science and Engineering, Shenyang University of Technology, Shenyang 110870, China; zhaolina@sut.edu.cn

**Keywords:** TIG welding, longitudinal magnetic field, high frequency, arc plasma, microstructure

## Abstract

This study proposes a novel high-frequency flat-top longitudinal magnetic field-assisted tungsten inert gas (HF-FTLMF TIG) welding method to improve arc constriction and weld penetration. The effects of magnetic field intensity and frequency on arc morphology, weld appearance, and microstructure were systematically investigated. The results show that, compared with conventional TIG welding, when the frequency of the FTLMF exceeds 1000 Hz, the arc becomes noticeably constricted, the conical angle decreases, and the heat input becomes more concentrated. Under appropriate magnetic field conditions, the arc pressure increases from 251.3 Pa for the free arc to 452.9 Pa, and the weld penetration depth increases from 0.84 mm to 1.09 mm. In addition, HF-FTLMF reduces surface unevenness and improves weld uniformity. Compared with low-frequency FTLMF (<1000 Hz), HF-FTLMF exhibits more stable arc behavior and better weld formation. SEM and EDS results further indicate that the application of HF-FTLMF affects the fusion-zone microstructure and elemental redistribution, and a relatively finer microstructure was observed under the 2000 Hz condition. These findings suggest that HF-FTLMF provides an effective approach for regulating TIG arc behavior and improving weld formation through magnetic field assistance.

## 1. Introduction

The demand for metal sheets, thin-walled welded pipes, and fire-resistant cables is steadily increasing in emerging industries such as electric vehicles and new energy grids [1,2,3]. Owing to its stable arc, spatter-free operation, and superior weld appearance, TIG welding has been widely employed in these applications [4,5,6]. To further enhance welding efficiency and joint quality, various arc-control techniques have been developed, including activated TIG (A-TIG) [7], keyhole TIG (K-TIG) [8], ultrasonic TIG (U-TIG) [9], magnetron TIG [10,11], and hybrid TIG [12]. A-TIG can effectively increase weld penetration by applying an activating flux to the workpiece surface; however, the additional flux-coating step is unfavorable for automated welding [13]. K-TIG provides a high heat input, but it is not suitable for thin-sheet welding [14]. U-TIG can regulate arc behavior and molten-pool flow through ultrasonic assistance; nevertheless, its modulation stability remains relatively poor, and intermittent excitation may occur during continuous operation, thereby limiting its practical application [15]. Hybrid TIG can further improve penetration and welding efficiency by coupling TIG with another heat source; however, its equipment configuration is relatively complex and costly [16].

By contrast, magnetron TIG has attracted considerable attention because of its non-contact nature, high flexibility, and excellent repeatability [17,18]. This technique improves weld quality by introducing an external magnetic field (EMF) during welding. According to the direction of the applied magnetic field, EMF-assisted TIG welding can generally be classified into longitudinal magnetic field (LMF), transverse magnetic field, cusp-shaped magnetic field, and rotating magnetic field [19,20,21]. Among these configurations, the LMF is one of the most widely adopted because it can regulate arc shape and may also influence weld microstructure, possibly through modifications of molten-pool flow under magnetic-field assistance [22,23].

Cui et al. observed that when the LMF is applied, the arc is compressed at the top and expands at the bottom, resulting in increased arc penetration [24]. Li et al. reported that LMF significantly reduces the intermetallic compound layer at the weld interface and enhances the tensile shear strength of the joint [25]. Chen et al. proposed an ultrasonic—axial magnetic field technique and found that the arc undergoes periodic compression and rotation; however, the degree of compression is less pronounced than that achieved with standing wave ultrasonic assistance [26]. Cunha et al. indicated that increasing the strength of the external magnetic field reduces the weld penetration depth, widens the weld seam, and refines the microstructure in the fusion zone [27]. Zhou et al. applied a composite magnetic field, consisting of LMF and a pointed magnetic field, and found comparable effects—decreased fusion depth, wider weld seams, and finer microstructures [28].

These findings suggest that a longitudinal magnetic field may improve certain weld characteristics through its influence on molten-pool flow, while the expansion at the arc bottom may contribute to a wider seam and shallower penetration [29,30]. Numerical simulations attribute this phenomenon to the rotation of charged particles caused by LMF, which expands the arc and generates a negative pressure region at the center [31,32]. This, in turn, leads to an upward gas flow through the arc core under centrifugal force—an effect described as anti-gravity flow [33,34].

To further improve the welding penetration and quality, an alternating longitudinal magnetic field (ALMF) has been introduced into the welding process. Mu et al. observed that applying ALMF produces a more uniform and refined fish-scale pattern on the weld surface and significantly reduces weld porosity [35]. Li et al. demonstrated that ALMF induces arc oscillation along the welding direction, leading to a narrower heat-affected zone and improvements in both strength and ductility of the weld [36]. Pu et al. reported that as the ALMF intensity increased from 0 to 60 mT, weld appearance improved; however, at 80 mT, arc stability and joint quality deteriorated, negatively affecting the weld appearance [37]. In narrow-gap laser-arc hybrid welding, the application of ALMF has been shown to significantly enhance the corrosion resistance of duplex stainless steel welds and reduce their susceptibility to stress corrosion cracking in seawater environments [38]. Moreover, introducing a sinusoidal alternating longitudinal magnetic field (SLMF) into K-TIG welding markedly enhances electromagnetic stirring in the molten pool [39], leading to refined microstructures and significantly improved impact toughness [40,41]. However, the limitations of magnetic field system power supplies restrict the frequency of ALMF, preventing deeper exploration of how magnetic field parameters affect welding performance.

Although previous studies have demonstrated that LMF can improve weld appearance and microstructure through electromagnetic stirring, most of these studies were conducted under static or low-frequency alternating magnetic fields. Under such conditions, the arc is prone to rotate and expand at its lower region, which often leads to a wider weld bead and limited penetration enhancement. Moreover, the frequency range of existing ALMF systems is largely constrained by the performance of magnetic field system power supplies, and therefore, the influence of high-frequency magnetic fields on arc compression and welding performance has not been systematically clarified. Furthermore, the use of the HF-FTLMF in TIG welding has not been reported.

In this study, a novel TIG welding method assisted by a high-frequency flat-top longitudinal magnetic field (HF-FTLMF) is proposed. By properly selecting the HF-FTLMF parameters, the arc can be effectively constricted, which is expected to promote higher arc pressure, deeper weld penetration, and microstructural refinement. Arc images obtained with and without HF-FTLMF are compared, and the effects of HF-FTLMF on arc characteristics, weld appearance, and microstructure are experimentally investigated.

## 2. Materials and Methods

### 2.1. Materials

The material used in this study was 316L austenitic stainless steel, with specific specifications of 220 mm × 60 mm × 6 mm. The chemical composition of the steel plate is shown in Table 1. The chemical composition listed in Table 1 is taken from the material certificate provided by the supplier. Slight variations may exist among different plates or batches. However, these small variations will not affect the conclusions of this study. Before the experiment, the test specimens were ground and polished to obtain a uniform surface finish. Subsequently, they were cleaned with anhydrous ethanol to remove residual contaminants and dried with compressed air. This pretreatment ensured adequate surface cleanliness for the welding experiments.

### 2.2. Experimental Parameters and Test Method

This study conducted TIG welding experiments using 316L austenitic stainless steel as the base metal, with a thickness of 6 mm. High-purity argon (99.99%, Ar 4.0) was used as the shielding gas at a flow rate of 16 L/min. The distance from the nozzle tip to the workpiece surface was set at 5 mm, and the welding torch travel speed was 0.3 m/min. The welding current was maintained at 100 A. Detailed welding conditions are provided in Table 2. To investigate the effects of the high-frequency longitudinal magnetic fields (HF-FTLMF) on arc behavior, molten metal flow, and joint performance, all welding parameters were held constant except for the HF-FTLMF parameters—specifically, the magnetic field frequency and peak-to-peak magnetic flux density. Six groups of experiments were conducted, with the corresponding HF-FTLMF parameters also listed in Table 3.

### 2.3. Experimental System and Equipment

**Figure 1:**
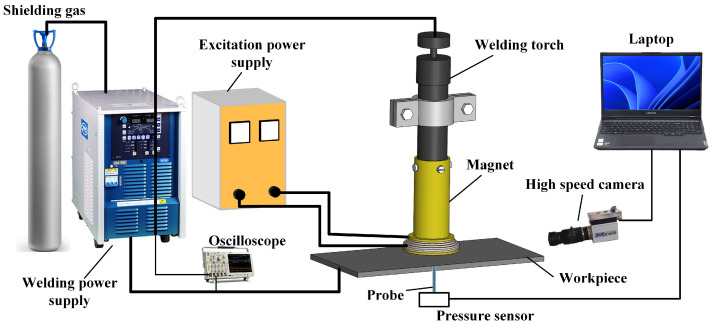
Schematic diagram of the HF-FTLMF TIG welding system.

The schematic diagram of the HF-FTLMF TIG welding system is shown in Figure 1. It mainly includes an automated TIG welding system (OTC-ADP400 TIG welding power supply, TIG welding torch, cooling water tank, mobile fixture, and welding platform), a self-developed magnetic field system (magnetic field system power supply, magnet), and a data acquisition system (Tektronix digital oscilloscope, high- speed camera, pickup coil).

#### 2.3.1. Welding System

**Figure 2:**
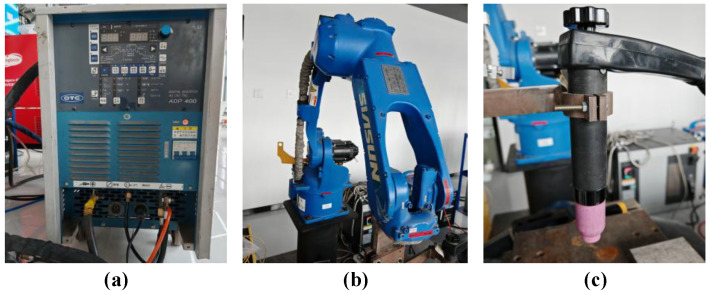
Main welding equipment used in the experiment: (**a**) TIG welding power source; (**b**) welding robot; (**c**) TIG welding torch.

The main welding equipment is shown in Figure 2. The welding system used in this study consisted of a robotic arm and an OTC-ADP400 TIG welding power supply. A specially designed fixture was mounted on the robotic arm to hold the welding torch, and the torch movement was controlled by the robot during welding. Before welding, the start point, end point, and travel speed were preset on the robot control panel. During the welding process, the torch was driven by the SR6CL welding robot to travel at a constant speed, as shown in Figure 2b.

#### 2.3.2. Magnetic Field System

The magnetic field system is composed of a magnet and a magnetic field system power supply. The magnet is an important component of the magnetic field system, and current passes through the magnet to generate a magnetic field. In order to generate a longitudinal magnetic field, the magnet in this article is designed as a helical tube. As shown in the Figure 1, the magnet is fixed to the welding gun through the magnet sleeve. In order to reduce the losses caused by skin effect caused by high-frequency current, the wire of the magnet is made of Litz wire—woven from multiple independently insulated thin copper wires.

**Figure 3:**
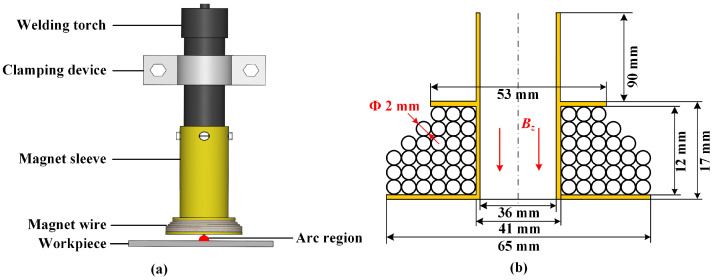
Schematic of HF-FTLMF magnet: (**a**) Magnet assembly; (**b**) Cross-section of magnet.

During the welding process, the distance between the magnet and the arc is very close, and the arc generates a large amount of heat. In order to keep the temperature of the magnet within a safe range, the magnet needs to minimize its own heating as much as possible. In order to generate a sufficiently strong magnetic field with minimal heat generation, the magnet needs to be optimized in design. After optimization design, the magnet has 30 turns and a wire diameter of 2 mm. Its structure is shown in the Figure 3. To verify the effectiveness of the design, a simulation model was built in COMSOL 6.0 to simulate the magnetic field and heat generation of the magnet, where the ambient temperature in the simulation is 293.15 K, with 5 m/s air-cooled heat dissipation. The COMSOL model was established based on the multiphysics coupling of Magnetic Fields, Laminar Flow, and Heat Transfer in Solids and Fluids. A transient simulation was performed to capture the time-dependent evolution of the physical fields. The computational mesh was generated using free triangular elements, with local mesh refinement applied in the magnet region to improve numerical accuracy. The minimum and maximum element sizes were 0.125 mm and 4.38 mm, respectively.

**Figure 4:**
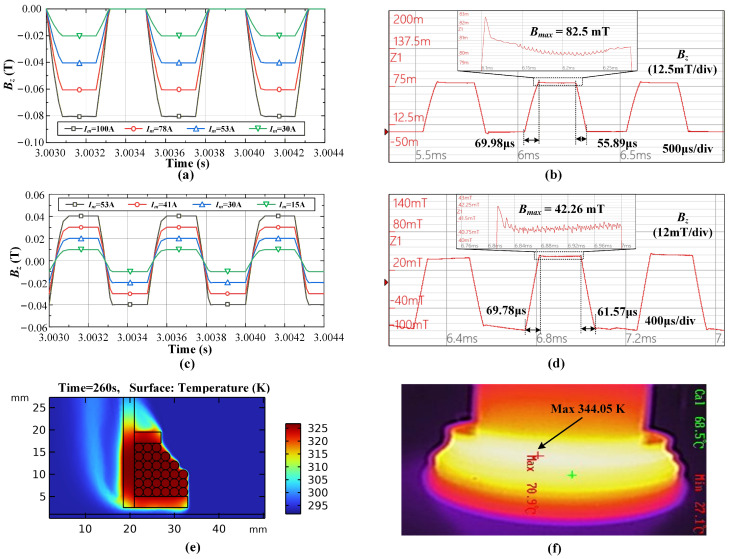
Comparison of simulation and experimental results of magnetic field system: (**a**,**b**) Unipolar HF-FTLMF; (**c**,**d**) Bipolar HF-FTLMF; (**e**,**f**) Magnet temperature.

Figure 4 illustrates the comparison of simulation and experimental results of the magnetic field system, where $I_{m}$ is the maximum magnet current and $B_{max}$ is the maximum magnetic flux density. The changing magnetic field will generate an induced voltage in a pickup coil, which can be recorded and integrated to reconstruct the magnetic field waveform. Figure 4a and Figure 4b show the simulated and experimental results of the unipolar HF-FTLMF, respectively, while Figure 4c and Figure 4d show the simulated and experimental results of the bipolar HF-FTLMF, respectively. The experimental results agree well with the simulation predictions. For the unipolar magnetic field, the measured peak magnetic flux density $B_{max}$ is 82.5 mT, with a relative error of only 3.1% compared with the simulated value of 80 mT at a magnet current of 100 A, and the rising edge is 69.98 μs, and the falling edge is 55.89 μs. For the bipolar magnetic field, the measured peak $B_{max}$ is 42.26 mT, with a relative error of only 5.6% compared with the simulated value of 40 mT at a magnet current of 53 A, and the rising edge is 69.78 μs, and the falling edge is 61.57 μs. In addition, the experimentally measured waveforms are highly consistent with the simulated results in terms of trapezoidal shape and the transient characteristics of the rising and falling edges, with no obvious waveform distortion or amplitude drift. These comparisons between simulation and experiment further verify the reliability of the magnetic field system design.

Figure 4e shows that the maximum temperature of the simulated magnet is 341.86 K, whereas the maximum temperature measured in the experiment is 344.05 K in Figure 4f. The slightly higher experimental value can be attributed to the insulation layer in the actual magnet, which impedes heat dissipation and is difficult to model accurately. Nevertheless, the close agreement between simulation and experiment verifies the reliability of the proposed method.

#### 2.3.3. Data Collection System

This experiment uses the FASTCAM-Ultima 512 high-speed camera. In order to test the accuracy of the shooting, a high-speed camera was mounted on the automatic welding equipment to ensure that the high-speed camera and the welding gun were relatively stationary during movement. The height of the high-speed camera and the welding gun were adjusted to be consistent, the aperture and focal length were adjusted, and the collected images were observed on the computer monitor.

The sensor measurement method is used to measure the distribution of arc pressure. A 230 mm × 150 mm × 18 mm copper plate is used to make a water-cooled copper anode. The water-cooled copper anode is hollow inside and has upward copper pipes on both sides of the upper end, serving as the inlet and outlet for water cooling and preventing the experimental device from being burned by the high-temperature TIG welding arc. There is a small downward hole located at the center of the water-cooled copper anode, one end of which is used to connect the CYG1103 piezoresistive pressure transmitter. At the same time, the pressure transmitter is connected to the SY-1C digital display to obtain the instantaneous arc pressure value.

The main equipment information mentioned above is summarized in Table 4, including the model, manufacturer, and country of origin of each device.

## 3. Results and Discussion

### 3.1. Arc Shape

**Figure 5:**
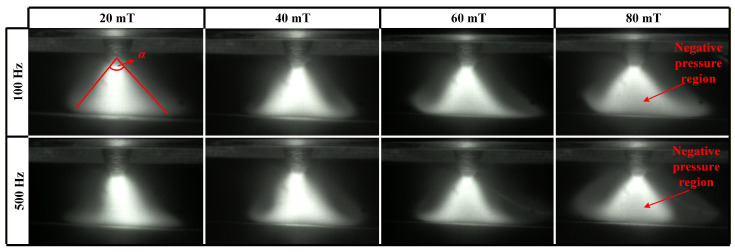
Arc shape under LF-FTLMF.

Figure 5 compares the arc shapes under various parameters of low-frequency FTLMF (LF-FTLMF). When the magnetic flux density of the applied LF-FTLMF (20 mT) is low, the magnetic field exerts only a minor influence on the arc, which appears slightly tilted—most likely due to magnetic blow caused by the grounding wire connection. As the magnetic flux density increases, the arc becomes noticeably broader. As the magnetic field strength increases further, the arc expands accordingly, and this morphological change is likely accompanied by a negative pressure region characterized by reduced brightness [33].

**Figure 6:**
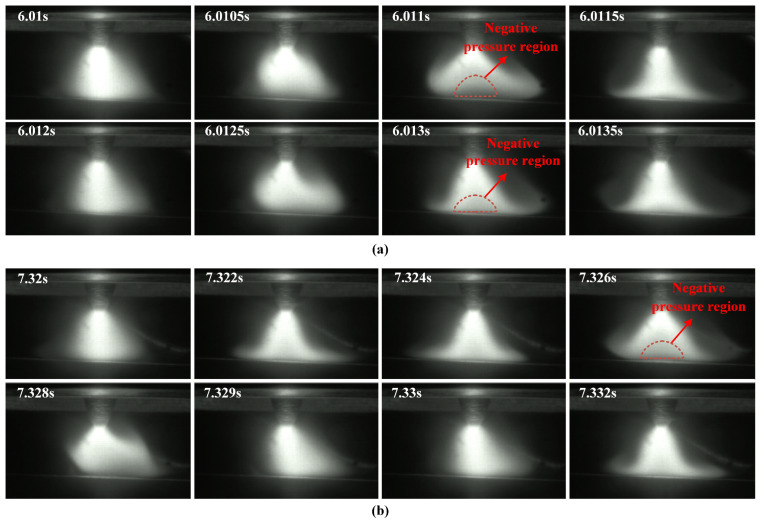
Instantaneous arc shape under LF-FTLMF: (**a**) 500 Hz; (**b**) 100 Hz.

Figure 6 shows the instantaneous arc evolution within a very short time window under LF-FTLMF conditions, in order to capture the transient variation of arc morphology during one magnetic-field cycle. As shown in Figure 6a, the arc shapes exhibit periodic variations under LF-FTLMF. At 500 Hz, an apparent low-brightness region, possibly associated with a negative-pressure region, appears during the arc expansion stage (6.01–6.011 s) and disappears during contraction (6.011–6.012 s). A similar behavior is observed at 100 Hz in Figure 6b, with the expansion and contraction cycles matching the period of the applied magnetic field. These results indicate that the arc variation period is synchronized with the external magnetic field, and the periodicity becomes more pronounced as the frequency decreases. The different arc shapes observed at 6.0125 s and 7.329 s are related to the different magnetic field states at these two moments. At 6.0125 s, the unipolar FTLMF enters the flat-top stage, where the magnetic flux density is maintained at a relatively stable level. Under this condition, the arc exhibits a more stable rotational behavior, and its morphology changes accordingly. The corresponding arc shape may be associated with the formation of a negative pressure region near the arc center. In contrast, at 7.329 s, the magnetic flux density of the unipolar FTLMF decreases to 0, and the arc returns to the free-arc state, resulting in a morphology similar to that without magnetic field assistance. Under these FTLMF conditions, the arc appears relatively unstable, and the observed morphology may be associated with the transient formation of a negative-pressure region near the arc center. This feature may contribute to a lower central arc pressure and, consequently, to shallower penetration. Moreover, increasing the magnetic flux density tends to intensify the periodic fluctuation of the arc, which may be unfavorable for weld quality.

**Figure 7:**
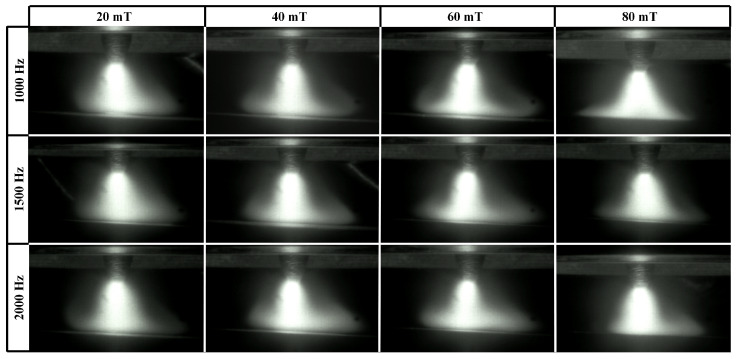
Arc shape under unipolar HF-FTLMF.

When the frequency of the FTLMF exceeds 500 Hz, the arc shapes under the unipolar HF-FTLMF are shown in Figure 7. As the frequency increases to 1000 Hz, the arc becomes constricted, and further increases in frequency lead to additional arc contraction. Compared with the LF-FTLMF, the tilt of the arc is significantly reduced. Under these HF-FTLMF parameters, the arc remains stable, and due to the constriction, the conical angle decreases, leading to a more concentrated arc heat. However, when the magnetic flux density of the HF-FTLMF is relatively small (20 mT), the compression effect of the external magnetic field is greatly weakened, resulting in only slight arc constriction. It is worth noting that when the magnet current reaches 106 A (corresponding to a magnetic flux density of 80 mT in the arc region), the arc slightly expands. Although the expansion at 2000 Hz is less pronounced than that at 100 Hz, it still reduces the heat flux density and consequently decreases the penetration depth. Within the tested parameter range, a peak magnetic flux density between 40 mT and 60 mT showed favorable arc constriction performance under unipolar HF-FTLMF.

**Figure 8:**
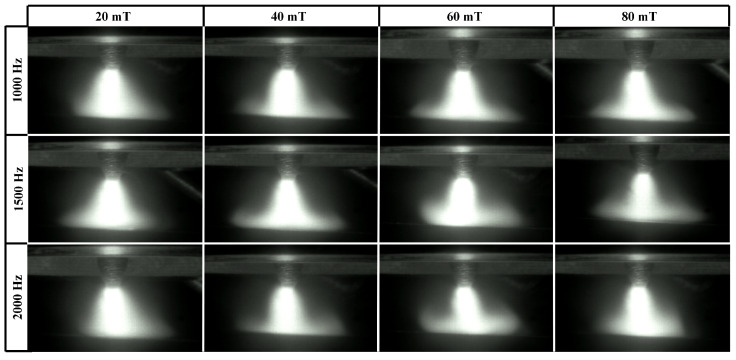
Arc shape under bipolar HF-FTLMF.

The arc shapes under a bipolar HF-FTLMF are shown in Figure 8. Similar to the unipolar HF-FTLMF, when the bipolar HF-FTLMF frequency reaches 1000 Hz, the arc becomes constricted, and further increases in frequency lead to additional arc contraction. When the peak-to-peak magnetic flux density of the bipolar HF-FTLMF is 40 mT or 60 mT, the arc remains stable, and due to the constriction, the conical angle decreases. However, unlike the unipolar HF-FTLMF, even when the magnetic flux density of the bipolar HF-FTLMF is smaller than 20 mT, the external magnetic field still exerts a noticeable constriction effect on the arc. Although the degree of constriction decreases, its effectiveness remains superior to that of the unipolar HF-FTLMF. Moreover, even when the peak-to-peak magnetic flux density increases to 80 mT, the arc remains stable, and the compression effect of the bipolar HF-FTLMF becomes most significant. Hence, for the bipolar HF-FTLMF, a higher peak-to-peak magnetic flux density can be applied to obtain a more pronounced arc constriction effect within the tested range.

The above analysis indicates that the arc shapes depend on both the frequency and the magnitude of the applied magnetic field. Arc stabilization and constriction require a frequency of at least 1000 Hz and a magnetic flux density higher than 20 mT. However, an excessively high magnetic flux density is not beneficial, as it may be accompanied by the appearance of a negative-pressure-like region in the arc. A possible explanation is that, during the flat-top stage, the relatively sustained magnetic constraint may favor a more pronounced unidirectional rotational tendency of the arc. Under such conditions, a vortex-like behavior may develop and may be associated with the emergence of a negative-pressure region. This interpretation is consistent with previous observations reported under sinusoidal high-frequency longitudinal magnetic fields, but it should be regarded as a literature-supported explanation rather than a directly demonstrated mechanism in the present study.

### 3.2. Arc Characteristics

**Figure 9:**
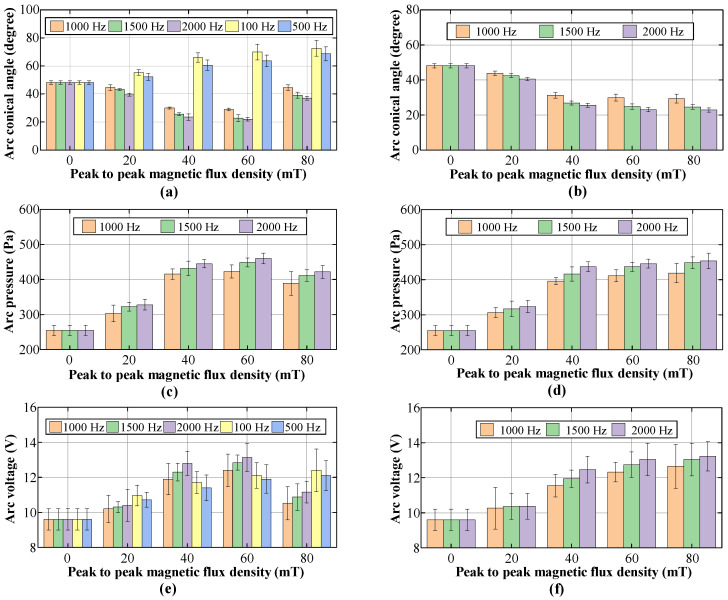
Arc characteristics at different frequencies: (**a**) Arc conical angle under unipolar FTLMF; (**b**) Arc conical angle under bipolar FTLMF; (**c**) Arc pressure under unipolar FTLMF; (**d**) Arc pressure under bipolar FTLMF; (**e**) Arc voltage under unipolar FTLMF; (**f**) Arc voltage under bipolar FTLMF.

To compare the effect of FTLMF on arc under different parameters, the arc voltage and arc pressure were measured under different FTLMF conditions. For each experimental condition, three repeated experiments were conducted, and in each experiment, five values were recorded and averaged. Figure 9 summarizes the arc characteristics under different FTLMF parameters.

Figure 9a,b compares the conical angles of the arc during welding under various FTLMF parameters. The conical angle of the arc is defined as the angle formed between the two boundary lines extending from the upper part of the arc to the bottom edge of the arc, as indicated by angle α, as illustrated in Figure 5. For each condition, the conical angle is measured from multiple arc images, and the average value is used. When the unipolar HF-FTLMF is applied, the conical angle of the arc varies as shown in Figure 9a. When the peak-to-peak magnetic flux density of the FTLMF at the arc region is 20 mT, the conical angles at all frequencies are nearly identical to those of the free arc. For FTLMFs above 1000 Hz, the arc slightly contracts, resulting in a smaller conical angle, whereas for frequencies below 1000 Hz, the arc slightly expands, showing a larger conical angle. As the peak-to-peak magnetic flux density increases to 40 mT, the influence of the HF-FTLMF on the arc becomes more pronounced. Under the HF-FTLMF above 1000 Hz, the arc exhibits a distinct contraction, and its conical angle decreases to nearly half that of the free arc, and the visible morphological change becomes relatively small with further frequency increase. In contrast, under the HF-FTLMF below 1000 Hz, the arc expands significantly, leading to a conical angle larger than that of the free arc. When the peak-to-peak magnetic flux density is further increased to 80 mT, the conical angle of the arc expands under unipolar HF-FTLMF, whereas it becomes further reduced under bipolar HF-FTLMF, as shown in Figure 9b.

Figure 9c,d illustrates the variation of arc pressure during TIG welding under different frequencies and magnetic flux densities. In conventional TIG welding without the external magnetic field assistance, the arc pressure is 251.3 Pa. When the HF-FTLMF is applied, the arc begins to constrict, and the arc pressure increases. As the peak-to-peak value of the magnetic flux density rises, the effect of the unipolar HF-FTLMF on the arc becomes more significant. Under unipolar HF-FTLMF, the arc pressure reaches its maximum (452.9 Pa) at 60 mT and subsequently decreases, as an excessively high magnetic flux density causes the arc to expand again, resulting in the reduction of arc pressure. In contrast, under bipolar HF-FTLMF, the arc pressure continuously increases with the peak-to-peak magnetic flux density, reaching its maximum (450.7 Pa) at 80 mT. Moreover, at the same peak-to-peak magnetic flux density, the arc pressure is higher under 2000 Hz HF-FTLMF. It can be seen that the arc pressure is highly sensitive to the maximum magnetic flux density; therefore, to achieve higher arc pressure, for unipolar HF-FTLMF, the peak magnetic flux density should preferably not exceed 60 mT. It should be noted that under 100 Hz and 500 Hz LF-FTLMF conditions, the arc is highly unstable, and a central low-pressure or negative-pressure-like region may appear during the expansion stage. However, the possible negative pressure cannot be measured accurately, because the CYG1103 pressure sensor has a measurement range of 0–10 kPa and cannot directly detect negative pressure. Therefore, the corresponding pressure values under 100 Hz and 500 Hz are not presented, and Figure 9c,d only show the arc-pressure results for FTLMF frequencies of 1000 Hz and above.

Figure 9e,f show the arc voltage signals measured during the welding process under different applied magnetic field parameters. In conventional TIG welding without LMF assistance, the arc voltage is 9.6 V. When FTLMF is applied, either at high or low frequency, the arc voltage increases compared with the free-arc condition; however, the underlying reasons differ. Under HF-FTLMF, the magnetic field constricts the arc, reduces the width of the conductive channel, and thereby increases the arc voltage drop. In contrast, under LF-FTLMF, the arc tends to expand, which increases the conductive path length and also results in a higher voltage drop. As the peak-to-peak magnetic flux density increases, the arc voltage under unipolar LF-FTLMF increases continuously. This trend is consistent with the more pronounced arc expansion at low frequency, where a stronger magnetic field further enhances the elongation of the conductive channel. Under unipolar HF-FTLMF, the arc voltage first increases and reaches a maximum value of 13.22 V, but begins to decrease when the peak-to-peak magnetic flux density rises to 80 mT, suggesting that the arc starts to re-expand and the conductive channel becomes wider. By contrast, under bipolar HF-FTLMF, the arc voltage continues to increase with increasing magnetic flux density and reaches 13.14 V, indicating that the alternating magnetic polarity is more favorable for maintaining arc constriction and stable energy concentration.

Overall, these results indicate that both the frequency and amplitude of the applied FTLMF have significant effects on the arc characteristics. Within the tested parameter range, 2000 Hz combined with a peak-to-peak magnetic flux density of 60 mT for the unipolar HF-FTLMF and 80 mT for the bipolar HF-FTLMF showed favorable overall performance. However, the present data are more sufficient to indicate a useful parameter range and an overall trend than to establish a statistically definitive optimal condition.

### 3.3. Weld Formation

**Figure 10:**
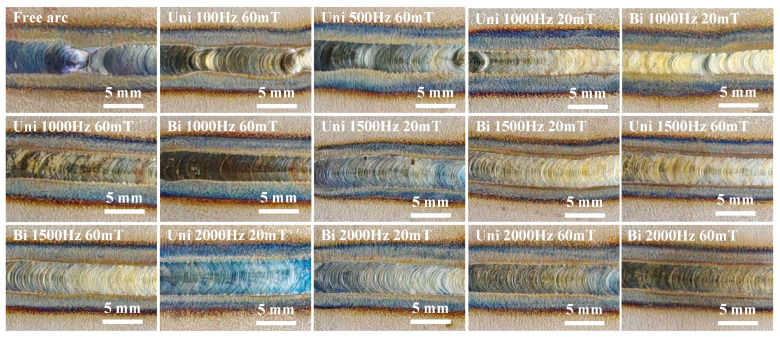
Macroscopic appearance of weld seam under different FTLMF.

Figure 10 illustrates the morphology of TIG welds under different FTLMF conditions. It can be observed that, without an external magnetic field, the weld exhibits an uneven surface morphology. When the applied magnetic flux density is 20 mT, the surface unevenness remains visible, indicating that the magnet current is too low to significantly affect the arc behavior and thus fails to markedly improve the weld quality.

However, when the magnetic flux density is increased to 60 mT, and the HF-FTLMF is applied, the arc stiffness is enhanced, and the weld morphology is notably improved. The weld exhibits a uniform, regular fish-scale pattern with a smooth surface, minimal spatter, and no observable hump defects. In contrast, under low-frequency magnetic field conditions, the weld width becomes uneven, and the hump defects become more pronounced. This may be related to the tendency of the low-frequency magnetic field to produce a hollow or low-brightness region in the central part of the arc, together with periodic arc oscillations, which may lower the central arc pressure and reduce arc stability. Conversely, the high-frequency magnetic field appears to exert a stronger constraining effect on the arc, thereby enhancing arc stiffness. In addition, the improved weld uniformity may also be related to changes in molten-pool flow behavior under magnetic-field assistance, although the present results do not directly demonstrate the stirring mechanism.

**Figure 11:**
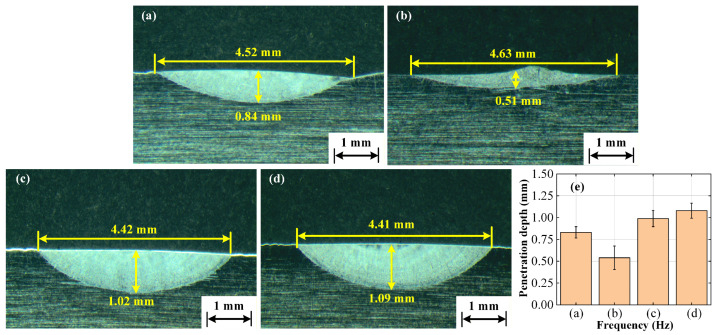
Cross-section morphology and penetration statistics of weld beads: (**a**) Free arc; (**b**) 100 Hz; (**c**) Bipolar HF-FTLMF (2000 Hz); (**d**) Unipolar HF-FTLMF (2000 Hz); (**e**) Average penetration depth with error bars obtained from repeated measurements.

The cross-sectional morphologies of TIG-welded joints under different flat-top longitudinal magnetic fields are shown in Figure 11. Under the free-arc condition (Figure 11a), a slight undercut can be observed at the weld edges. The weld surface width, penetration depth, and cross-sectional area are 4.52 mm, 0.84 mm, and 2.06 mm^2^, respectively. Under the 100 Hz low-frequency FTLMF condition (Figure 11b), the weld width increases slightly to 4.63 mm, whereas the penetration depth and cross-sectional area decrease to 0.51 mm and 0.89 mm^2^, respectively. Compared with the free-arc condition, the width increases by 2.43%, while the penetration depth and cross-sectional area decrease by 39.29% and 56.80%, respectively. This indicates that the low-frequency magnetic field mainly promotes arc spreading and lateral heat distribution, resulting in a wider and shallower weld. By contrast, under the unipolar HF-FTLMF condition (Figure 11d), the weld surface width, penetration depth, and cross-sectional area are 4.41 mm, 1.09 mm, and 2.61 mm^2^, respectively, corresponding to a width reduction of 2.43% and increases in penetration depth and cross-sectional area of 29.76% and 26.70%, respectively. Under the bipolar HF-FTLMF condition (Figure 11c), the corresponding values are 4.42 mm, 1.02 mm, and 2.47 mm^2^, respectively, indicating a width reduction of 2.21% and increases in penetration depth and cross-sectional area of 21.43% and 19.90%, respectively. These results show that, under the same peak magnetic flux density, the unipolar HF-FTLMF exhibits a more pronounced penetration enhancement effect than the bipolar HF-FTLMF.

To improve the reliability of the penetration evaluation, repeated measurements were performed for each condition. Specifically, three repeated welding experiments were conducted, and the penetration depth was measured at three positions for each weld bead. The average values and corresponding error bars are presented in Figure 11e. The results show that the penetration under the low-frequency condition is lower than that of the free arc, whereas the penetration tends to increase with increasing frequency within the tested range. Under the present experimental conditions, the HF-FTLMF-assisted cases exhibit greater average penetration than the free-arc condition.

It can be seen that the application of HF-FTLMF significantly increases the weld penetration compared with the case without a magnetic field, while the application of a LF-FTLMF produces a wider and shallower weld. This phenomenon may be related to the hollow or negative-pressure-like region that tends to appear under low-frequency alternating magnetic field conditions, which may lower the central arc pressure and alter the heat-transfer pattern, thereby causing more heat to accumulate near the molten-pool surface [34]. In contrast, under HF-FTLMF, the arc is observed to become more constricted, accompanied by increased arc pressure and a more concentrated heat input, which is likely associated with the increase in weld penetration.

**Figure 12:**
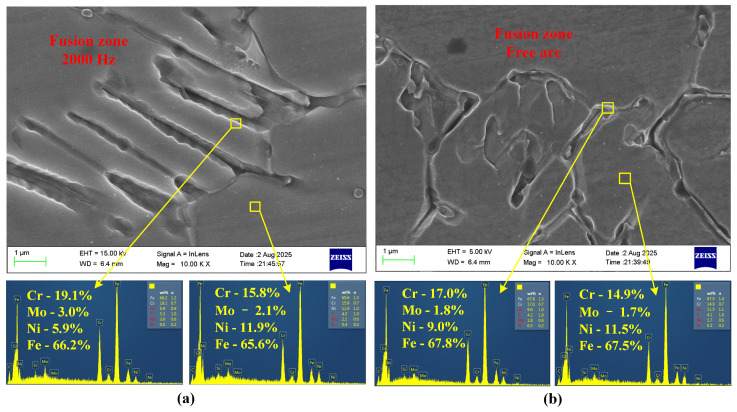
SEM and EDS analysis of fusion zone under HF-FTLMF (2000 Hz) and free arc: (**a**) 2000 Hz HF-FTLMF; (**b**) Free arc.

The microstructures of the fusion zone (FZ) of AISI 316L stainless steel under the unipolar HF-FTLMF condition and the free-arc condition are shown in Figure 12. For austenitic stainless steel welds, the phase constitution of the fusion zone is closely related to the solidification conditions and the $Cr_{eq}/Ni_{eq}$ ratio. The values of $Cr_{eq}$ and $Ni_{eq}$ were calculated according to Equations (1) and (2). In general, a higher $Cr_{eq}/Ni_{eq}$ ratio corresponds to a stronger ferrite-forming tendency and a lower tendency for austenite formation during solidification.

(1) $${Cr}_{eq}=\mathrm{Cr}\%+\mathrm{Mo}\%+1.5\times \mathrm{Si}\%+0.5\times \mathrm{Nb}\%,$$

(2) $${Ni}_{eq}=\mathrm{Ni}\%+30\times C\%+0.5\times \mathrm{Mn}\%.$$

The EDS results further reveal the elemental partitioning behavior in the fusion zone. The selected region with higher contents of Cr and Mo but a lower content of Ni may indicate a relatively stronger ferrite-forming tendency, whereas the region with a significantly higher Ni content and lower Cr and Mo contents may indicate a relatively stronger austenite-forming tendency. However, these EDS results only provide indirect compositional support and do not constitute direct phase identification. This elemental distribution is broadly consistent with compositional trends commonly associated with the enrichment of ferrite-forming elements such as Cr and Mo and austenite-forming elements such as Ni during solidification. Nevertheless, the present EDS results alone are insufficient for definitive phase identification or for establishing a firm solidification-mode interpretation.

As shown in Figure 12a and Figure 12b, the SEM micrographs and corresponding EDS spectra represent the fusion-zone microstructures obtained under the 2000 Hz HF-FTLMF condition and the free-arc condition, respectively. The results show that the fusion-zone microstructure under 2000 Hz HF-FTLMF is relatively finer, denser, and more elongated, whereas that under the free-arc condition is comparatively coarser and more loosely distributed. This suggests that the high-frequency magnetic field may affect molten-pool convection and solidification behavior, which may contribute to the observed microstructural differences. The EDS results further indicate that the contents of Cr and Mo in the fusion zone under the magnetic-field-assisted condition are higher than those in the free-arc specimen. For example, the Cr and Mo contents in the selected region under 2000 Hz HF-FTLMF reach 19.1 wt.% and 3.0 wt.%, respectively, compared with about 17.0 wt.% and 1.8 wt.% in the free-arc condition. These results suggest that HF-FTLMF influences solute redistribution during solidification and thereby affects the microstructure and elemental distribution in the fusion zone.

**Figure 13:**
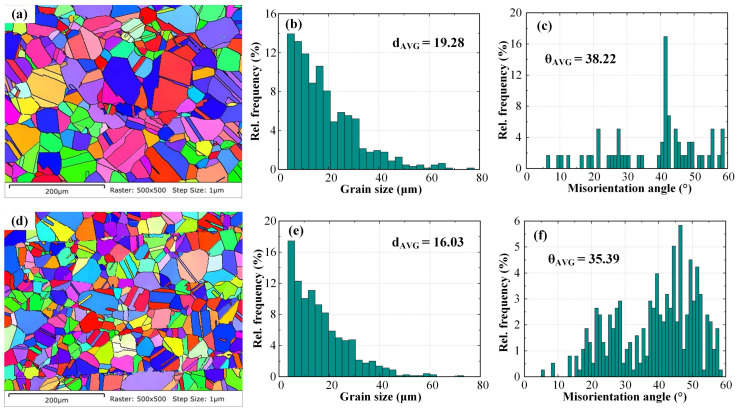
Weld seam center EBSD maps and grain size analysis: (**a**) weld seam center without magnetic field, (**b**) weld seam center with alternating magnetic field, (**c**) distribution of misorientation angles in weld seam center without magnetic field, (**d**) distribution of misorientation angles in weld seam center with alternating magnetic field, (**e**) grain size distribution in weld seam center without magnetic field, (**f**) grain size distribution in weld seam center with alternating magnetic field.

EBSD was employed to characterize the size, type, and morphology of the central grains in the weld seam. Figure 13 presents the average grain size and morphology at the weld seam center for TIG-welded joints with and without HF-FTLMF assistance. Near the center of the molten pool, the crystallization rate is the highest due to the minimal temperature gradient, high solute concentration, and pronounced undercooling. Consequently, numerous equiaxed grains are distributed in the weld seam center. The crystallographic orientation at the center is relatively uniform, showing no evident texture, with different orientations randomly distributed. As shown in Figure 13a,b, several fine grains can be observed at the weld center, which may result from recrystallization. Figure 13c,d show that, without magnetic field assistance, the average grain size at the center of the TIG-welded joint is approximately 19.28 μm, whereas under a high-frequency alternating magnetic field it decreases to about 16.03 μm, representing a 16.86% reduction compared with the condition without magnetic field assistance. Figure 13e,f illustrate the grain boundary distribution and the corresponding misorientation angle proportions in the two regions. Grain boundaries between 2° and 5° are classified as low-angle boundaries, while those above 15° are defined as high-angle boundaries. Without HF-FTLMF assistance, the proportion of low-angle boundaries at the weld center is 6.7%, and the average misorientation angle is 38.22°. With the application of an alternating magnetic field, these values change to 1.6% and 35.39%, respectively. The variation in the proportion of low-angle grain boundaries may be related to changes in microstructural evolution during solidification; however, direct evidence for grain-boundary migration or dynamic recrystallization is not provided in the present study. This result suggests that the high-frequency alternating magnetic field may influence molten-pool flow and solidification behavior, which could enhance the fluidity of the liquid metal and contribute to the increase in the proportion of high-angle grain boundaries. A magnetic-field-assisted stirring effect may be involved, but this mechanism is not directly demonstrated in the present study. With increasing FTLMF frequency, the influence on grain refinement appears to become more pronounced. The present EBSD results mainly indicate an observed microstructural tendency in the analyzed region under the present experimental conditions, rather than a universally conclusive grain-refinement effect.

### 3.4. Mechanism Analysis

**Figure 14:**
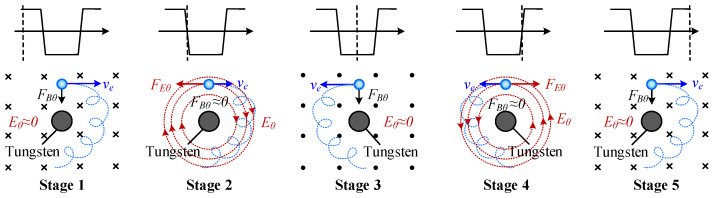
Schematic illustration of the mechanism by which the HF-FTLMF influences the arc behavior through electromagnetic forces acting on electrons.

Figure 14 illustrates the force analysis of electrons observed above the cathode under the influence of the FTLMF, where $F_{B\theta }$ denotes the circumferential Lorentz force, $F_{E\theta }$ represents the circumferential electric force, $v_{e}$ is the electron velocity, and the blue circles indicate electrons. The blue dashed line indicates the trajectory of electron motion. In TIG welding, the tungsten electrode is connected to the negative terminal of the power supply, while the workpiece is connected to the positive terminal. Consequently, the arc mainly consists of electrons emitted from the tungsten electrode. Therefore, the discussion focuses on the motion of electrons within the arc to explain the mechanism by which the FTLMF influences the arc behavior. As shown in Figure 14, the circumferential electric field $F_{E\theta }$ changes direction with the variation of the FTLMF. When the FTLMF frequency is high, during the flat-top stage of the magnetic field (stage 1), the charged particles in the arc are constrained by the magnetic field. However, since the duration of the flat-top stage is very short, the arc cannot form a stable unidirectional rotation. During the transition stage of the magnetic field (stage 2), the circumferential electric field induced by the HF-FTLMF can further reduce the rotational velocity of the arc. In contrast, under LF-FTLMF, the flat-top duration of the magnetic field is relatively long. During stage 1, the arc is more likely to exhibit a stable unidirectional rotational tendency under the applied magnetic field, which may promote a vortex- or tornado-like behavior and may be associated with the formation of a negative-pressure region. However, this should be regarded as a plausible interpretation based on previous studies and the observed arc morphology, rather than a directly verified mechanism in the present work.

The HF-FTLMF waveform obtained experimentally in Section 2 is subjected to a Fourier transform, and its Fourier-expanded expression can be written as:

(3) $$B_{FT}=\frac{a_{0}}{2}+\sum_{n=1}^{\infty }\left[a_{n}\cos\left(n\omega_{p}t\right)+b_{n}\sin\left(n\omega_{p}t\right)\right],$$

where $\omega_{p}$ is the angular frequency of HF-FTLMF, equals to $2\pi f_{p}$. $a_{0}$, $a_{n}$, and $b_{n}$ are the Fourier coefficients of HF-FTLMF, and their expressions are given by:

(4) $$a_{0}=\frac{B_{max}\left(t_{r}+t_{f}\right)}{T_{p}},$$

(5) $$a_{n}=\frac{8B_{max}}{T_{p}t_{r}n^{2}\omega_{p}^{2}}\sin\left(\frac{n\omega_{p}t_{r}}{2}\right)\sin\left(\frac{n\omega_{p}\left(t_{r}+t_{f}\right)}{2}\right)\cos\left[n\omega_{p}\left(t_{r}+\frac{t_{f}}{2}\right)\right],$$

(6) $$b_{n}=\frac{8B_{max}}{T_{p}t_{r}n^{2}\omega_{p}^{2}}\sin\left(\frac{n\omega_{p}t_{r}}{2}\right)\sin\left(\frac{n\omega_{p}\left(t_{r}+t_{f}\right)}{2}\right)\sin\left[n\omega_{p}\left(t_{r}+\frac{t_{f}}{2}\right)\right],$$

where $T_{p}$, $t_{r}$, and $t_{f}$ are the period of the HF-FTLMF, rise time, and flat-top time of the HF-FTLMF, respectively.

**Figure 15:**
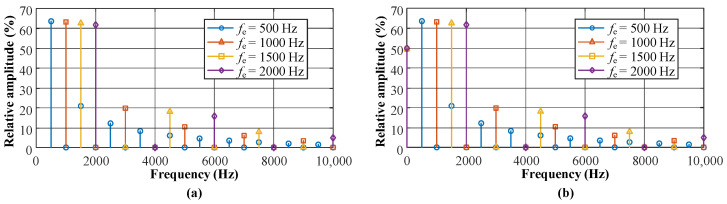
Spectrum diagram of the HF-FTLMF with different frequencies: (**a**) Bipolar HF-FTLMF; (**b**) Unipolar HF-FTLMF.

In the actual calculation process, in order to reduce the computational complexity while ensuring sufficient accuracy, the Fourier series is approximated using a finite number of terms. In this study, n=10 is adopted to calculate and analyze the major spectral components. Therefore, the frequency spectrum of HF-FTLMF at different frequencies $f_{p}$ can be obtained, as shown in Figure 15.

It can be seen from Figure 15 that the HF-FTLMF contains multiple frequency components. The DC component provides a relatively stable magnetic constraint on the arc plasma, which helps maintain the overall magnetic compression effect. In contrast, the high-frequency components keep the arc in a rapid commutation state, so that the charged particles do not remain in a single rotational mode for a long period. This dynamic behavior may help suppress the formation of a negative-pressure region that is more likely to occur under a relatively steady magnetic constraint [42]. Under the combined action of the DC component and the high-frequency components, the arc tends to become more constricted and stable, which is likely associated with higher arc pressure and more concentrated heat input [39]. As a result, the weld penetration is enhanced. However, when the peak-to-peak magnetic flux density increases to 80 mT, the amplitude of the DC component in the unipolar HF-FTLMF also increases. Under the stronger DC component, the arc may again exhibit a more pronounced unidirectional rotational tendency, which could contribute to arc re-expansion [33]. By contrast, the bipolar HF-FTLMF does not contain a DC component. Therefore, even when the peak-to-peak magnetic flux density reaches 80 mT, the arc does not show the same re-expansion behavior as in the unipolar case, and the constriction effect can still be maintained.

## 4. Conclusions

A high-frequency flat-top longitudinal magnetic field (HF-FTLMF) assisted TIG welding method was proposed. TIG welding was performed on 6 mm-thick 316L stainless steel plates with the assistance of HF-FTLMF. The effects of HF-FTLMF on the arc shape, macroscopic weld characteristics, microstructure, and grain size were investigated in detail. The conclusions are summarized as follows:

(1) HF-FTLMF was found to significantly constrain and stabilize the welding arc, producing a more concentrated and stiffer arc column. Compared with conventional TIG welding, the arc under HF-FTLMF exhibited higher arc pressure and a larger arc voltage drop, which suggests enhanced arc constriction and stability.
(2) The application of HF-FTLMF was associated with increased arc pressure and more concentrated heat input, and the average weld penetration increased by 29.7% under the selected condition compared with conventional TIG welding. These results suggest that within the investigated conditions, HF-FTLMF can effectively improve penetration under the present experimental conditions.
(3) In addition, EBSD results from the analyzed region suggest a tendency toward grain refinement under HF-FTLMF assistance. Compared with conventional TIG welding, the average grain size under HF-FTLMF was reduced by 16.86%. The observed refinement may be related to changes in molten-pool flow and solidification behavior under magnetic-field assistance, although the detailed mechanism requires further direct verification.

## Figures and Tables

**Table 1:** Chemical composition of the steel plate (wt.%).

Elements	C	Si	Mn	P	S	Cr	Ni	Mo	Fe
Content	0.03	0.40	1.32	0.045	0.021	16.0	10.9	2.1	Bal.

**Table 2:** Welding process parameters.

Welding Parameters	Value
Welding current	100 A
Welding speed	0.3 m/min
Shielding gas (flow rate)	High-purity argon (16 L·$\min^{-1}$)
Electrode-workpiece distance	5 mm

**Table 3:** Experimental groups.

Group	Frequency (Hz)	Peak-to-Peak Magnetic Flux Density (mT)	Polarity
1	free arc	0	-
2	100	80/60/40/20	Unipolar
3	500	80/60/40/20	Unipolar
4	1000	80/60/40/20	Bipolar/Unipolar
5	1500	80/60/40/20	Bipolar/Unipolar
6	2000	80/60/40/20	Bipolar/Unipolar

**Table 4:** Main equipment used in the experiment.

Equipment	Model	Manufacturer	Country
Welding power source	OTC-ADP400	WID	China, Suzhou
High-speed camera	FASTCAM Ultima 512	PHOTRON	Japan, Osaka
Arc pressure sensor	CYG1103	JCSENSOR	China, Xi’an
Welding robot	SR6CL	SIASUN	China, Shenyang
Magnetic field system	Self-developed	Huazhong University of Science and Technology	China, Wuhan
Oscilloscope	MDO3014	Tektronix	USA, OR, Beaverton

## Data Availability

The original contributions presented in this study are included in the article. Further inquiries can be directed to the corresponding authors.
